# Mutated Fanconi anemia pathway in non-Fanconi anemia cancers

**DOI:** 10.18632/oncotarget.4056

**Published:** 2015-05-09

**Authors:** Yihang Shen, Yuan-Hao Lee, Jayabal Panneerselvam, Jun Zhang, Lenora W. M. Loo, Peiwen Fei

**Affiliations:** ^1^ Program of Cancer Biology, University of Hawaii Cancer Center, University of Hawaii, Honolulu, HI, USA; ^2^ Department of Laboratory Medicine and Pathology, Mayo Clinic, Rochester, MN, USA; ^3^ Program of Epidemiology, University of Hawaii Cancer Center, University of Hawaii, Honolulu, HI, USA

**Keywords:** Fanconi anemia genes, TCGA, the mutated FA pathway, tumorigenesis, cancer treatment

## Abstract

An extremely high cancer incidence and the hypersensitivity to DNA crosslinking agents associated with Fanconi Anemia (FA) have marked it to be a unique genetic model system to study human cancer etiology and treatment, which has emerged an intense area of investigation in cancer research. However, there is limited information about the relationship between the mutated FA pathway and the cancer development or/and treatment in patients without FA. Here we analyzed the mutation rates of the seventeen FA genes in 68 DNA sequence datasets. We found that the FA pathway is frequently mutated across a variety of human cancers, with a rate mostly in the range of 15 to 35 % in human lung, brain, bladder, ovarian, breast cancers, or others. Furthermore, we found a statistically significant correlation (*p* < 0.05) between the mutated FA pathway and the development of human bladder cancer that we only further analyzed. Together, our study demonstrates a previously unknown fact that the mutated FA pathway frequently occurs during the development of non-FA human cancers, holding profound implications directly in advancing our understanding of human tumorigenesis as well as tumor sensitivity/resistance to crosslinking drug-relevant chemotherapy.

## INTRODUCTION

Germline mutations in both alleles of a Fanconi Anemia (FA) gene lead to FA, a rare human genetic disease, which is also referred to a chromosomal abnormality syndrome [1-5]. Homozygous germline mutations in each individual FA gene account for a corresponding FA complementation group. Common features shared among all complementation groups indicate that encoded proteins function in a similar or common signaling transduction pathway, named the FA pathway or the FA-BRCA pathway, in regard to the direct or indirect involvement of breast cancer susceptibility genes, *BRCA1* and *BRCA2* [6-8]. We recently reported that the functional heterozygosity occurring in the FA signaling pathway during the course of cancer development plays a crucial role in promoting the development of human cancer in patients without FA [9, 10]. These studies, for the first time, demonstrated the tumor suppressor role of the FA signaling pathway predicated in 1971 by Dr. Swift [11]. To date, there are seventeen FA genes (*FANC/A, B, C, D1, D2, E, F, G, I, G, L, M, N O, P, Q and S)* that have been identified [6, 7] and the functions of these genes have emerged as an intense area of investigation in cancer research [6, 9, 10, 12, 13]. It is not too hard to recognize how important this signaling pathway is in enhancing our understanding of human tumorigenesis. However, the relevant knowledge of how the genetically mutated FA pathway is involved in the development of non-FA human cancers remains to be limited. Here we report common occurrences of the mutated FA pathway and its strong association with the development of human bladder cancer that was only further analyzed. This study, for the first time, demonstrated the importance of the FA tumor suppressor pathway at the genetic level among the general population.

## RESULTS

The recent technological advances offer us research tools that are much more articulated than what we could imagine before. Whole metabolome, transcriptome or genome analysis is of growing importance in advancing our views on the cancer development and treatment [14-16]. However, the annotation of the relationship between the mutated FA pathway and human cancer in a genome-wide manner is lacking. Here we analyzed a total of 68 publicly available DNA sequence datasets for mutations occurring in the 17 FA genes and compounded a sum rate for the mutated FA pathway via c-BioPortal [17, 18]. These sum rates are scattered from 1 to 55% with a frequency in a range of 15-35% (Table 1 and Supplementary Table 1). This is the first report to comprehensively show the scale of detectable mutations in the FA pathway in non-FA human cancers, firmly supporting our prior report that the FA tumor suppressor pathway plays a crucial role in suppressing cancer development in the patients without FA [9, 10]. Again, for the first time at the genetic level, the frequently mutated FA pathway as indicated (Table 1 and Supplementary Table 1) conveys its essential nature in suppressing the development of human cancers in the general population. Mutations occurring in any of the FA genes certainly contribute to the genetic hetero- or homo-zygosity of a given individual FA gene, and hereafter compromise the FA tumor suppressor signaling and promote the development of non-FA human cancer.

**Table 1:** The rates of the mutated FA pathway in non-FA human cancers

Tumors (sequence datasets from TCGA)	Percent of Cases Carrying Mutated Pathway	No. of Cases Sequenced
Acute myeloid Leukemia	4.30%	188
Adrenocortical Carcinoma	26.10%	88
Bladder Urothelial Carcinoma	52.80%	127
Brain Lower Grade Glioma	11.90%	286
Breast Invasive Carcinoma	36.10%	962
Cervical Squamous Cell Carcinoma and Endocervical Adenocarcinoma	23.60%	191
Colorectal Adenocarcinoma	24.10%	220
Esophageal Carcinoma	26.60%	184
Glioblastoma Multiforme	12.80%	273
Head and Neck Squamous Cell Carcinoma	34.40%	302
Kidney Chromophobe	10.60%	66
Kidney Renal Clear Cell Carcinoma	20.70%	415
Kidney Renal Papillary Cell Carcinoma	16.10%	161
Liver Hepatocellular Carcinoma	29%	193
Lung Adenocarcinoma	39%	172
Lung Squamous Cell Carcinoma	50.60%	178
Lymphoid Neoplasm Diffuse Large B-cell Lymphoma	27.10%	48
Ovarian Serous Cystadenocarcinoma	46.60%	311
Pancreatic Adenocarcinoma	35.60%	90
Pheochromocytoma and Paraganglioma	7.50%	161
Prostate Adenocarcinoma	25.20%	258
Sarcoma	29.60%	257
Skin Cutaneous Melanoma	44.20%	278
Stomach Adenocarcinoma	39.10%	220
Thyroid Carcinoma	5%	399
Uterine Carcinosarcoma	28.60%	56
Uterine Corpus Endometrial Carcinoma	36.40%	242

**Figure 1:**
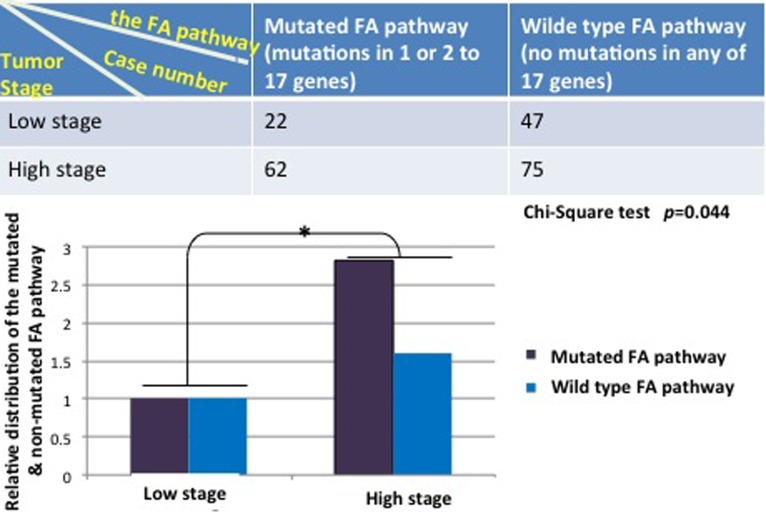
The mutated FA pathway is associated with the development of human bladder cancer On the basis of the clinic notes available for a set of human bladder cancer samples on TCGA, we divided this set of cancer samples into two groups with or without a mutated FA pathway. We further divided each group into two subgroups upon tumor stages. We combined cases at the clinic stage 0, I, and II as the low stage, and those at the clinic stage III and IV as the high stage considering the limited sample sizes. The top table shows the distribution of bladder cancer cases in the groups we defined. The relative distribution of the mutated or non-mutated FA pathway in bladder cancer was plotted in bars at the bottom to suggest the role of a mutated FA pathway is statistically significant in promoting tumor growth from the low stage to the high stage in comparison with the wild type FA pathway. Chi-square test was performed with a *p* value = 0.044.

**Figure 2:**
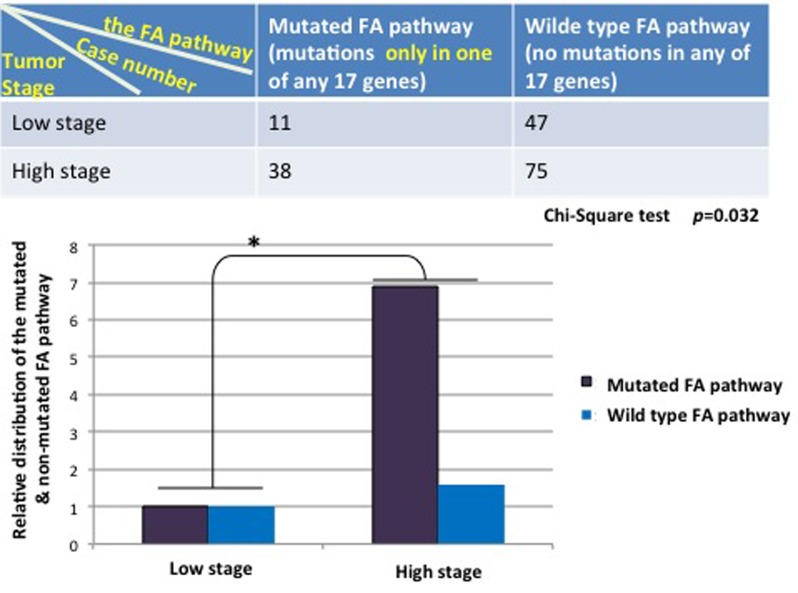
The mutated FA pathway resulting only from one mutated FA gene appears to potently promotes the development of human balder cancer Bladder cancer cases were regrouped according to the mutated FA pathway harboring only one of the mutated FA genes, accompanying the same criterion used previously (Figure 1) for the subgroups. The top table shows the distribution of bladder cancer cases in each group we redefined. The relative distribution of the mutated or non-mutated FA pathway in bladder cancer was plotted in bars at the bottom to suggest the role of a single mutated FA gene-containing pathway is statistically significant in promoting tumor growth from the low stage to the high stage in contract to the wild type status of the FA pathway. Chi-square test was conducted with a *p* value = 0.032.

To further understand the importance, at the genetic level, of those detectable mutations in the FA pathway in contributing to human tumorigenesis among the general population, we asked how significant the rate of the mutated FA pathway is during the development of human bladder cancer by cross-referencing the available clinical information, provided by the Cancer Genome Atlas (TCGA). We defined the mutated FA pathway, resulting from mutations found in one or 2 to 17 FA genes known so far; and the corresponding wild type FA pathway, meaning that no mutations are found in any of the 17 FA genes. We manually extracted the patient information of bladder tumor stages from the clinic notes available on TCGA. As shown in Figure 1, the mutated FA pathway, in contrast to the non-mutated one, is significantly correlated with the tumor promotion with a *p* value = 0.044 (more tumor cases at the high (late) stage when the FA pathway is mutated), indicating the tumor suppressor role played by the non-mutated FA pathway during the development of human bladder cancer. Next, we suspected this correlation might be heavily attributed to the mutated pathway resulting from multiple mutated FA genes, not the sole influence of the mutated FA pathway, noting that each FA gene has multiple functions in addition to its role in the FA pathway [19-22]. We regrouped cases by counting the mutated FA pathway resulting from a single mutated FA gene, and found that this redefined mutated FA pathway remains to be significantly correlated with tumor promotion (*p* = 0.032) (Figure 2). This further indicates the importance of the FA tumor suppressor pathway during the development of human bladder cancer. Our studies on human bladder cancer once again genetically validate that the FA tumor-suppressor signaling is not restricted to the FA cells.

## DISCUSSION

We here demonstrate a previously unrecognized fact that the mutated FA pathway frequently occurs in the non-FA human tumors (Table 1 and Supplementary Table 1). We analyze the frequency of mutations to FA genes in the FA pathway, by including point mutations, deletions, as well as gene amplification events present in *FANC/A, B, C, D1/2, E, F, G, I, G, L, M, N O, P, Q or S* [6, 7]. The latter type of mutation might be subject to a point relevant to an over-activating FA pathway, rather than the loss of function. However the reported studies suggested that the over-activated FA signaling is tumorigenic [13, 23] and, thus, generally leads to the loss of tumor suppressive effect as a point mutation or deletion would. As a matter of fact, such scale rates of the mutated FA pathway occurring in human cancers ascertain extreme importance of the FA tumor suppressor pathway; once mutated, it promotes the development of human cancer as indicated in Figures 1 and 2. Our studies and many others [9, 19, 24-26] showed that somatic inactivation of the FA pathway could be at the level of any FA proteins or others functionally relevant. The compromised FA signaling pathway, if including epigenetic modifications of the FA genes [27] and other factors directly or indirectly on FA proteins [9, 28], would have a much higher rate in non-FA cancer patients than those accordingly shown in Table 1 and Supplementary Table 1 only on the basis of mutations in the 17 FA genes.

The irreparable, accumulated DNA damage contributes to the development of human cancer; on the other hand, those accumulated DNA damage resulting from cancer therapeutic agents [29-33], would be beneficial to the outcome of cancer treatment. FA cells carrying a defective FA pathway are sensitive to DNA crosslinking agents and die over time [1-5]. Therefore, tumor cells, harboring a mutated FA pathway, and eventually carrying accumulated DNA damage, will die after the exposure to therapeutic drugs [12, 34]. Consequently, the genetic profiling of FA genes may be a strategy to predict the sensitivity of cancer treatment. This would be very helpful for avoiding the drawbacks of general therapies in clinic. For instance, Bacillus Calmette-Guerin (BCG) [35-37] is given to patients with non-muscle-invasive bladder cancer mainly as immunotherapy without prior knowledge of clear functioning mechanisms. Ideally, BCG would be administered only to those patients who do not carry the mutated FA signaling and to give platinum-related drugs to patients who would have functional hetero- or homo-zygosity of the FA pathway (FA gene mutations, oncogenic factors compromising FA signaling, etc.). Looking at the explicit rates of the mutated FA pathway in all types of human cancers (Table 1 and Supplementary Table 1), there is a very low rate (<5%), for example, in medulloblastoma. This could be a rational explanation for the unclear benefit reported for medulloblastoma chemotherapy in using a combination including cisplatin or carboplatin [38]. Because of the low rate of the mutated FA pathway in medulloblastoma (1.8 or 4.3%, calculated from two separate datasets) (Supplementary Table 1), most tumor cells carrying a fully functioning FA pathway genetically commit to repair the damaged DNA caused by cisplatin or carboplatin and presumably continue to proliferate.

The field of FA cancer research progresses rapidly, and there are FA cases yet to be defined [39]. This may raise an issue of how to accurately define the wild type status of the FA pathway. We could have counted the cases harboring mutations in any of unidentified FA genes for the wild type group (Figures 1 and 2). However, the wild type status of the FA pathway named upon no mutation in any of the 17 FA genes would be the closest condition we can possibly provide as far (Main functions of the 17 genes outlined in Supplementary Table 2). Most importantly, there is a statistically significant correlation between the mutated FA pathway and the tumor promotion of human bladder cancer in the settings we studied (Figures 1 and 2), supporting the genetic evidence that the FA pathway has a role as a tumor suppressor in non-FA individuals and further concurring with our studies in this exciting field [9, 10, 12, 13, 21, 40-43]. While we had an in-depth look at the mutated FA pathway causing from only one mutated FA gene (Figure 2), we wanted to see how the mutated FA pathway resulting from two or more mutated FA genes is related to the tumor promotion. Unfortunately, it did not show statistical significance in terms of tumor promotion potentiated by the mutated FA pathway, but remains to hold a higher tendency of promoting tumor growth as compared to the wild type pathway (2.4 folds versus 1.6 folds for tumors at the high/late stage carrying the mutated or non-mutated status of the pathway, respectively). This may result from a limited sample size or/and the state of tumors that would have entered into the phase of the highest malignancy at the genetic level. In addition, owing to a tremendous amount of time needed in manually extracting clinical information, we did not go on performing similar analyses for other types of cancers in this study. We believe the finding would be similar to what shown in Figures 1 and 2, indicating the importance, at the prospective of cancer genetics [44, 45], of the tumor suppressor role played by the FA pathway during the development of non-FA human cancers. Of note, we realized that c-BioPortal does not give the clear information whether the output mutations affect both alleles or/and the all functions of any given gene. Possibly, the given gene may have a certain level of normal functions though mutated. Nonetheless, either heterozygous or homozygous mutations occurring in FA genes will compromise the tumor suppressor function of the FA signaling pathway and promote the development of human cancers. In fact, this has been evidenced by numerous studies reporting an increased incidence of breast, ovarian, pancreatic, or prostate cancer tightly associated with the heterozygosity of several FA genes (FANCD1, FANCJ, FANCN, FANCO or/and FANCS) [22, 46-57].

## MATERIALS AND METHODS

### Publicly available DNA sequence datasets

A total of 68 DNA sequence datasets were used and analyzed upon the known 17 FA genes as far via c-BioPortal [17, 18] to determine a mutation rate of the FA pathway in a variety of human cancers. The sources of those sequence datasets are TCGA, MSKCC, BGI, BCCRC, Nature, Nature Genetics, Cell, Science, AMC, Cancer Cell, and PNAS. The rates of the mutated pathway were calculated upon 27 sequence datasets of TCGA (Table 1), and others on the basis of 41 sequence datasets available in the rest of resources herein stated (Supplementary Table 1).

### Source of clinic information of human bladder cancer samples

The clinic information for a total of 206 human bladder cancer cases was downloaded from TCGA database (updated on Jan 17, 2015). The related information for tumor stage according to The American Joint Committee On Cancer (AJCC) was collected manually and plotted as described in the text.

### Statistical analysis

The correlation between the mutated FA pathway and the cancer stage was analyzed using Chi-square test. A *p*-value <0.05 was considered as statistical significance. All analyses were processed by SPSS 20 software.

## SUPPLEMENTARY TABLE


