# Supplementary material for: Mutated Fanconi anemia pathway in non-Fanconi anemia cancers
Source: Oncotarget. 2015 May 9;6(24):20396–403. doi: 10.18632/oncotarget.4056 (PMC4653013; doi:10.18632/oncotarget.4056)
Supplement: Supplementary file 1 [file oncotarget-06-20396-s001.pdf]

Mutated Fanconi anemia pathway in non-Fanconi anemia cancers

Supplementary Material

Supplementary Table 1

Tumors	Percent of Cases Carrying Mutated Pathway	No. of Cases Sequenced
Adenoid Cystic Carcinoma (MSKCC, Nature Genetics 2013)	20%	60
Bladder Cancer (MSKCC, Eur Urol 2014)	12.80%	109
Bladder Cancer (MSKCC, JCO 2013)	13.40%	97
Bladder Urothelial Carcinoma (BGI, Nature Genetics 2013)	15.20%	99
Breast cancer patient xenografts (British Columbia, Nature 2014)	69%	29
Breast Invasive Carcinoma (British Columbia, Nature 2012)	7.70%	65
Breast Invasive Carcinoma (Broad, Nature 2012)	7.80%	103
Breast Invasive Carcinoma (Sanger, Nature 2012)	18%	100
Cancer Cell Line Encyclopedia (Novartis/Broad, Nature 2012)	54.30%	881
Colorectal Adenocarcinoma (Genentech, Nature 2012)	27.80%	72
Colorectal Adenocarcinoma Triplets (MSKCC, Genome Biology 2014)	2.20%	138
Esophageal Adenocarcinoma (Broad, Nature Genetics 2013)	13.70%	146
Esophageal Squamous Cell Carcinoma (ICGC, Nature 2014)	9.10%	88
Head and Neck Squamous Cell Carcinoma (Broad, Science 2011)	21.60%	74
Head and Neck Squamous Cell Carcinoma (Johns Hopkins, Science 2011)	3.10%	32
Intrahepatic Cholangiocarcinoma (Johns Hopkins, Nature Genetics 2013)	2.50%	40
Kidney Renal Clear Cell Carcinoma (BGI, Nature Genetics 2012)	3.70%	81
Liver Hepatocellular Carcinoma (AMC, Hepatology 2014)	12.60%	231
Lung Adenocarcinoma (Broad, Cell 2012)	30.20%	182
Lung Adenocarcinoma (TSP, Nature 2008)	1.80%	163
Malignant Peripheral Nerve Sheath Tumor (MSKCC, Nature Genetics 2014)	33.30%	15
Medulloblastoma (Broad, Nature 2012)	4.30%	92
Medulloblastoma (ICGC, Nature 2012)	1.80%	114
Melanoma (Broad/Dana Farber, Nature 2012)	40%	25
Multiple Myeloma (Broad, Cancer Cell 2014)	5.40%	205
Nasopharyngeal Carcinoma (Singapore, Nature Genetics 2014)	5.40%	56
NCI-60 Cell Lines (NCI, Cancer Res. 2012)	49.10%	53
Pancreatic Adenocarcinoma (ICGC, Nature 2012)	2%	99
Prostate Adenocarcinoma (Broad/Cornell, Cell 2013)	17.90%	56
Prostate Adenocarcinoma (Broad/Cornell, Nature Genetics 2012)	4.60%	109
Prostate Adenocarcinoma (MSKCC, Cancer Cell 2010)	12.60%	103

Supplementary Table 1 (continued)

Tumors	Percent of Cases Carrying Mutated Pathway	No. of Cases Sequenced
Prostate Adenocarcinoma CNA study (MSKCC, PNAS 2014)	1%	104
Prostate Adenocarcinoma, Metastatic (Michigan, Nature 2012)	37.70%	61
Sarcoma (MSKCC/Broad, Nature Genetics 2010)	12.60%	207
Skin Cutaneous Melanoma (Broad, Cell 2012)	32.20%	121
Skin Cutaneous Melanoma (Yale, Nature Genetics 2012)	23.10%	91
Small Cell Lung Cancer (CLCGP, Nature Genetics 2012)	17.20%	29
Small Cell Lung Cancer (Johns Hopkins, Nature Genetics 2012)	11.90%	42
Stomach Adenocarcinoma (Pfizer and UHK, Nature Genetics 2014)	10%	100
Stomach Adenocarcinoma (U Tokyo, Nature Genetics 2014)	6.70%	30
Stomach Adenocarcinoma (UHK, Nature Genetics 2011)	27.30%	22

Supplementary Table 2. Summary of main known functions of the 17 FA genes

FANCA	FANCA participates in DNA repair and can be activated as a scaffold protein with FANCG in FA core complex via ATR phosphorylation (J Clin Invest. 2015;125(4):1523-32. Blood. 2009;113(10):2181-90.).
FANCB	FANCB, along with FANCL and FAAP100 forms one of the FA core complex and stabilizes FANCL for FANCD2 and FANCI mono-ubiquitination (Nat Genet. 2004;36(11):1219-24).
FANCC	FANCC and FANCE compose one of the subunits of the FA core complexes. In addition to DNA repair, FANCC also involves in apoptosis, redox regulation and cytokine signaling (Blood. 2003;101(10):3877-84. Mol Cell 2004;15(4):607-20.).
FANCD1 (BRCA2)	BRCA2 binds the single strand DNA and directly interacts with the recombinase RAD51 to stimulate strand invasion, a vital step of homologous recombination. The localization of RAD51 to the DNA double-strand break requires the formation of BRCA1-PALB2-BRCA2 complex. Davies OR, Pellegrini L. Nature Structure & Molecular Biology 2007, 14 (6). Buisson R, Masson J-Y. Nucleic Acids Research 2012, 1-12.
FANCD2	The mono-ubiquitination of FANCD2 is the most important step of FA pathway activation. FANCD2-FANCI complex recruits downstream nucleases for DNA repair. Wang AT, Smogorzewska A. SnapShot: Cell. 2015; 160: 354-354 e351
FANCE	FANCE and FANCC compose one of the subunits of the FA core complex. The repeated helical hairpins of this protein indicate its potential function of an ubiquitin ligase for FANCD2 ubiquitination (Annu Rev Biophys. 2014;43:257-78. Nucleic Acids Res. 2007;35(5):1638-48. EMBO J. 2002;21(13):3414-23).
FANCF	FANCF acts as an adaptor to hold all subunits of FA core complex together (Nat Genet. 2000;24(1):15-6).
FANCG	FANCG and FANCA compose one of the subunits of the FA core complex. The tetratricopeptide repeat motif of itsamino acid structure suggests a potential role as a scaffold in the core complex (DNA Repair (Amst). 2004;3(1):77-84.).
FANCI	Mono-ubiquitination modified FANCI and FANCD2 can form an I-D complex and translocate to damaged DNA to recruit DNA damage response proteins (Cell. 2007;129(2):289-301).
FANCJ (BRIP1)	FANCJ interacts with FANCS via the BRCT domain and translocates to damaged DNA. It acts as a DNA helicase to remodel the DNA structure (Nat Genet. 2005;37(9):934-5. Annu Rev Genet. 2009;43:223-49).
FANCL	FANCL,FANCB, FAAP100 constitutes a subunit of FA core complex and exerts the activity of anE3 ubiquitin ligase to induce FANCD2 and FANCI mono-ubiquitination (Nat Genet. 2003;35(2):165-170. Hum Mutat. 2009;30(7):E761-E770).
FANCM	FANCM-FAAP24 complex is able to recognize and translocate to damaged DNA for recruiting FA core complex to chromatin in the S phase of cell cycle (Blood. 2008;111(10):5215-22. Genes Dev. 2009;23(5):555-60).
FANCN (PALB2)	FANCN coordinates FANCD1 for chromatin localization (Mol Cel. 2006;22(6):719-29).
FANCO (RAD51C)	FANCO is recruited by FANCD1 to double-strand (Nature. 2010;467(7316):678-83) or single-strand (Nat Struct Mol Biol 2010;17(10):1263-5) DNA.
FANCP (SLX4)	FANCP's zinc finger structure is able to enhance the activity of endonucleases, such as MUS81 and MSH2/3, for DNA repair in mismatch repair (Proc Natl Acad Sci USA. 2011;108(16):6492-6. PLoS Genetics. 2011;7(6):e1002094). ERCC1-FANCQ/FANCP complex can remove damaged DNA bases via nucleotide excision repair pathway (Biochemistry. 2010;49(26):5560-9). FANCP-SLX1 complex can also resolve the Holliday Junction to keep chromatin replicate and separate correctly in homologous recombination repair (Cell. 2009;138(1):63-77).
FANCQ (ERCC4)	FANCQ, along with FANCP, SLX1 and ERCC1, exerts the function of flap endonuclease in nucleotide excision repair and DNA interstrand crosslinks repair (Am J Hum Genet. 2013;92(5):800-6. J Biol Chem. 1997;;272(6):3833-7).
FANCS (BRCA1)	BRCA1 participates in homologous recombination upon ATM activation. The polyubiquitin chains recruit BRCA1 to damaged DNA sites and facilitate its association with Mre11-Rad50-Nbs1 (MRN) complex, BRCA2-Rad51 complex, and BACH1 (also called BRIP1 or FANCJ)-TopBP1 complex. Zhang J. Cell & Bioscience 2013, 3:11; Zhang J, et al. and Xia F. Molecular and cellular biology 2004, 24(2):708-18; Lee, A Y-L, Wu X. The Journal of Biological Chemistry 2008, 283: 7713-7720; Cantor S B, Guillemette S. Future Oncology 2011, 7(2): 253-261.
